# Research progress of functional near-infrared spectroscopy in patients with psychiatric disorders

**DOI:** 10.1080/20961790.2020.1720901

**Published:** 2020-05-05

**Authors:** Fan Chang, Haozhe Li, Shengyu Zhang, Chen Chen, Chao Liu, Weixiong Cai

**Affiliations:** a Shanghai Key Lab of Forensic Medicine, Key Lab of Forensic Science, Ministry of Justice, Shanghai Forensic Service Platform, Academy of Forensic Science, Shanghai, China; b School of Mental Health, Wenzhou Medical University, Wenzhou, China

**Keywords:** Forensic sciences, psychiatric disorder, functional near-infrared spectroscopy, traumatic brain injury

## Abstract

Functional near-infrared spectroscopy (fNIRS) is a technique of detecting cerebral cortical function by using near-infrared light, which is a multifunctional neuroimaging technique and provides a convenient and efficient detection method in neuroscience. In consideration of acceptability, safety, high spatial and temporal resolutions compared with electroencephalogram (EEG) and functional magnetic resonance imaging (fMRI), fNIRS is widely used to study different psychiatric disorders, most prominently affective disorders, schizophrenic illnesses, brain organic mental disorders and neurodevelopmental disorders, etc. The article focuses on the latest research progress and practical application of fNIRS in psychiatric disorders, especially traumatic brain, including studies on the characterization of phenomenology, treatment effects and descriptions of neuroimaging data.

## Introduction

Psychiatric disorders have become a complex social and public health problem and create a new consensus on a global scale. According to relevant data [1], the rates of psychiatric illness are very high. The various psychiatric disorders will become more prominent after the 21st century. In the prediction of diseases in 2020, severe psychiatric diseases rank first in the total burden. For a long time, patients with psychiatric disorders face more physical, psychological, social and economic pressure than other patients, which brings serious economic burden and hidden danger to patients’ families and society. At present, most neuropsychiatric diseases are diagnosed depending only on their clinical symptoms, lacking objective neuroimaging biomarkers. It cannot provide individual-level diagnosis and prediction, and its clinical application value is very limited [2].

Functional near-infrared spectroscopy (fNIRS) is a relatively new technology and can provide a real-time understanding of the brain function state. Ehlis et al. [3] had concluded there were at least 115 original studies that had employed fNIRS to investigate psychiatric research questions in 2014. It is a portable, inexpensive and user-friendly device which is easily adapted to the outpatient setting for the assessment of cognitive functions, which other functional imaging technologies are not. Its loose applicability and high ecological validity make fNIRS particularly suitable for psychiatric patients who may fear stressful environments (e.g. in magnetic resonance imaging/position emission tomography (MRI/PET) scanners) or display motor restlessness (e.g. in attention deficit/hyperactivity disorder (ADHD)) by interfering with motion sensing such as MRI methods, electroencephalogram (EEG), magnetoencephalogram (MEG) or PET. Meanwhile, it has higher spatial and higher temporal resolutions in functional brain imaging than EEG and functional magnetic resonance imaging (fMRI), respectively, thus can make up for the shortcomings and interpret the research objectives more comprehensively [4]. Most of researches combine fNIRS with other brain imaging methods, such as Doppler sonography [5,6], fMRI [7–17], EEG [7,8,15–17], PET [18], and single photon emission computed tomography (SPECT), led to an amazing increase in studies implementing simultaneous applications. Despite some remaining shortcomings, including being easy to be affected by extracranial signals and anatomical parameters, fNIRS exhibits a number of important advantages making its application attractive for both neuroscience in general and psychiatric research particularly [19]. Thus, combining different imaging techniques, fNIRS can make an important contribution to the overall understanding of the functional characteristics underlying psychiatric disorders. On the other hand, fNIRS is able to assess cortical hemodynamics in the event that other methods fail (e.g. when there is a high requirement for whole-body movement in the subject). In summary, the advantages of fNIRS in psychiatric disorders are not only due to its relative insensitivity to movement artifacts, but also further related to its ease of use and high generality. Due to frequently repeated measurements, fNIRS can be easily used in longitudinal studies, which is becoming increasingly important in investigating the development and treatment of mental disorders.

## Basic principles and advantages of fNIRS

The fNIRS is a multifunctional neuroimaging technique that uses near-infrared light to detect cerebral cortical function [20]. Near-infrared light has strong penetrability to organisms. Main substances that absorb light are oxygenated haemoglobin (HbO) and deoxygenated haemoglobin (HbR), with different absorption rates in specific bands (about 760 nm and around 850 nm) in the spectral window (600 – 900 nm).

In the active cortical region, the concentration of HbO in the haemoglobin is increased and that of HbR is decreased [21]. According to this characteristic, near-infrared light can be directly irradiated to the surface of the scalp, and the intensity of scattered light in the cerebral cortex can be measured and utilized. Using Beer-Lambert law, one can complete the conversion of raw optical data to blood oxygen concentration to achieve simultaneous multi-point measurement of HbO and HbR concentration changes in specific regions and infer the changes in blood oxygen and blood volume in this brain region. Therefore, it is possible to locate and measure the functional activation of the local cerebral cortex and to study the functional connectivity of the brain by using fNIRS.

The fNIRS was first used in animal cerebral cortex blood oxygen level testing in 1977 [3,4], and then it received extensive attention in the biomedical community. In the early stages of the study, it is only used in the study of animal or infant cerebral cortical functional activities [22]. After 1991, thanks to the development of electronic computer and optical theory, it could be used to determine the motion law and distribution of light in tissues accurately [23]. In this way, it is possible to apply near-infrared imaging technology for the detection of brain function in adults. For the mysterious organ of the human brain, the introduction of fNIRS has created new opportunities for studying the neural processes in the human cerebral cortex [19].

## Schizophrenia and schizophrenia-like illness with fNIRS

The first study of fNIRS was published in 1994 of patients with schizophrenia (SZ). A split-personality questionnaire (SPQ) was used to examine the relationship between split-personality traits in non-clinical female groups with prefrontal activation patterns in the verbal fluency task (VFT) [24]. Compared with healthy subjects, the frontal lobe haemodynamic response was slower in schizophrenics with a steeper (slope) HbO concentration at the start of the task [25]. In addition, fNIRS tested brain activity in patients with SZ during a go/no-go mission, as well as their behaviour performance, was shown. It was found that SZ patients had a lower degree of activation on the frontal and temporal regions. Patients with SZ showed less activation in the superior and orbital frontal and middle temporal regions during the emotional go/no-go block [26]. Knowing the interaction between inhibition, the results indicated that frontotemporal dysfunction was associated with the pathophysiology of emotional-cognitive disorders in SZ patients [24,25,27]. Overall, these studies were performed during various neurocognitive tasks (e.g. random number generation, Hanoi Tower, Stroop, language fluency testing, go/no-go tasks). The abnormal brain activation patterns in patients with SZ were detected, especially in the prefrontal cortex (PFC) [28]. In almost all cases, reduced haemodynamic responses were observed in the frontal region of SZ patients compared with the control group [29], even when the sample’s age, sex and pre-disease IQ were statistically consistent with other neuroimaging methods for the detection of cerebral ischemia [30]. In addition to these studies on different executive functions, studies have consistently demonstrated the forehead defects of the SZ patients [31].

## Depressive disorders and bipolar disorders with fNIRS

Regarding the study of fNIRS in affective disorders, Okada et al. [32] conducted the first study (1996) focusing on the frontal lobe of patients with major depressive disorders (MDD) during the mirroring task. After this early work, frontal lobe abnormalities in depression were repeatedly mentioned in many fNIRS studies. Then more studies reported a reduction in bilateral anterior and posterior oxygenation times in patients with depression during the VFT [33–35]. Even more amazing is the study of healthy subjects in different emotional states, during the oral working memory (WM) task [36] or the N-Back task [37], where the relative concentration of oxyhaemoglobin in the prefrontal lobe was reported to be increased. Also, studies have also discussed the effects of some treatments on patients within prefrontal functions [36,38,39]. In addition, in 2000, researcher began to use fNIRS to study the clinical effects of repeated transcranial magnetic stimulation (rTMS) in the left dorsal lateral prefrontal cortex (DLPFC) of MDD patients [26].

The fNIRS has also been used to study bipolar disorder. In the resting time of WM tasks, compared with health controls, patients exhibited significantly reduced intra-regional and symmetrically interhemispheric connectivity in the PFC and decreased activity in the forehead, which could be the central neuroimaging markers of bipolar disorder [40]. But during the tasks related to attention and nonverbal cognition, on the contrary, the results showed an increase in ventral lateral dorsolateral prefrontal and frontal cortex activity in subjects and spatial visual WM storage [41]. We speculate that it may be the differences in cognitive tasks that lead to the different results. It may be that the tasks involve a wide range of prefrontal areas, and the differences in the areas of interest in the study cannot cover all of them. Of course, these findings suggest that fNIRS has the potential advantage of higher temporal resolution and can be used to investigate patients with different affective disorders, which may provide new opportunities for diagnosis.

## Other mental disorders with fNIRS

The fNIRS study on anxiety has so far focused on panic disorder and dental phobia. The fNIRS studies of panic disorder have shown that the activation of left PFC is reduced during general cognitive activation [42]. The patients with panic disorder showed hypofrontality including the DLPFC via fNIRS, and verum intermittent theta burst stimulation (iTBS) could not augment prefrontal activity, nevertheless, the increased activation in the left inferior frontal gyrus has been found after sham iTBS [43]. In a large sample of 109 panic patients, task-related oxygenation can be further demonstrated. In most cases, the significant correlation between change and severity of symptoms has been shown, demonstrating that left forehead HbO is inversely related to panic attack frequency while the right forehead HbO is associated with severity of phobia [44,45].

Patients with post-traumatic stress disorder (PTSD) showed a significantly smaller response of HbO and total hemoglobin in the verbal fluency task compared with health controls via fNIRS and had significantly lower attention and concentration scores in the Wechsler Memory Scale-Revised, which was positively correlated with the increase of total hemoglobin in the verbal fluency task. Pre-frontal dysfunction in patients with PTSD may be a secondary phenomenon in reducing attention [46]. In summary, these studies have shown that PTSD patients exhibit significant cerebral haemodynamic changes in the face of trauma-related substances, and the frontal dysfunction during the cognitive tasks generally seems to be the inevitable result of the major changes in attention [47].

For one of the most frequent personality disorders, studies investigated frontal lobe function and social exclusion experience related to mood regulation. Patients with a social anxiety disorder (SAD) experienced abnormal fear in normal social situations. The result showed that the left frontal cortex was active in SAD patients [44]. In addition, when studying borderline personality disorder (BPD) while viewing sad picture data, it was found that BPD patients had a reduced slope associated with task-related increases in the left prefrontal channel compared to controls [48]. On the contrary, in the experience of social exclusion, it was found that BPD patients showed increased activation in the frontal-polar cortex [49], indicating that different neural mechanisms are the basis of different symptom dimensions. Certain personality traits have been shown to be associated with changes in cortical HbO concentration.

In the fNIRS study on addiction, Schecklmann et al. [50] studied three patient groups (acute withdrawal, detoxification and moderation) during the VFT and found generally reduced cerebral oxygenation levels in the frontotemporal region. From alcohol dependence to abstinence to normal function, during Iowa Gambling Test (IGT), Wisconsin Card Sorting Test (WCST) and N-Back, the performance of multi-substance users was worse than that of the individually matched control group. During the IGT process, they also showed less dorsolateral prefrontal oxidative. The results showed that cognitive decline was observed even after controlling for impulsivity in college students who took multiple drugs. This confirms the suitability of fNIRS to study treatment effects in both longitudinal and acute challenge environments.

## Neurodevelopmental disorders with fNIRS

ADHD is a neurodevelopmental disorder prevalent in children. Neuroimaging studies have revealed neurological abnormalities in some areas of the brain, including the frontal cortex, striatum, cerebellum and occipital cortex. One study used permutation entropy (PE) to measure fNIRS in the complexity of WM task signals in children with ADHD and found that the PE value of the right dorsolateral PFC was positively correlated with the ADHD index [51]. These results suggest that the complexity analysis of fNIRS signal may be a promising tool for the diagnosis of children with ADHD. In addition, an fNIRS study recently explored the neurocognitive effects of methylphenidate (MPH) in children with ADHD during the attentional task and behavioural control paradigm [52].

In addition, autism spectrum disorder (ASD) constitutes another developmental pathology that has been frequently studied using fNIRS in the past few years. Most of these investigations are directed to prefrontal haemodynamics, which are studied in letter fluency testing. Children with ASD had a decrease in leftward bias of the balance between right and left rostral prefrontal cortex (RoPFC) activity when compared with normally developing children via fNIRS [53]. In addition, the test was also performed in the unaffected siblings of participants with ASDs. The results of the assessment suggest a change in age-related prefrontal lobe activity in ASD and the effect of genes on this phenomenon [54].

## Traumatic brain injury and organic mental disorders with fNIRS

Traumatic brain injury is common in human beings. The disease can be discriminated by comparing the absorption of brain tissue on both sides. A large number of studies have also confirmed the clinical application values of fNIRS in the diagnosis and dynamic monitoring of cerebral ischemia and haemorrhagic diseases [55]. fNIRS has been used to evaluate the neurological changes in individuals with traumatic brain injury (TBI) during Stroop tasks [56,57]. The results showed that fNIRS could identify the common frontal lobe inefficiencies in TBI [58] that were the same as using fMRI [59]. Also, there is a study on the cortical activation and connectivity patterns in visual attention processing after TBI in adolescents. Focusing on the sports-related young adult concussion (SRC), the results showed that SRC group had significantly increased brain activity in the left medial frontal gyrus (MFG) and enhanced functional connectivity between the right suboccipital cortex and bilateral Calgary gyrus (CG). Interestingly, it was also found that the amount of MFG activation on the left side was significantly negatively correlated with the severity of hyperactive/impulsive symptoms in the normal control group, but not related to the patient [60]. However, in most training tasks, the change of oxyhaemoglobin in patients with TBI tends to be higher than that in the medial frontal area, and the change of oxyhaemoglobin in the two tasks in the medial frontal area is significantly different. This suggests that fNIRS measurements are also useful in assessing changes in neural activity during rehabilitation tasks in patients with TBI [61]. In a word, in studies of TBI, almost all of the focus areas are located in the prefrontal lobe and the results are often consistent with studies such as fMRI and CT, and it can make up for some of the technology’s shortcomings.

Imaging of life-long brain development is not only limited to the examination of developmental disorders in children and young adults but also in depicting the neurophysiological functions of the elderly. For the importance of age-related cortical haemodynamic effects in cognitive tasks, elderly subjects, compared to the young adults, had reduced HbO concentrations during VFT without left cerebral activation [62]. In a large sample of 325 healthy older adults, cortical oxidative activity decreased in the inferior frontal lobe junction but increased in the middle frontal lobe, indicating compensatory processes or cortical recombination [63]. Oxygenation in patients with Alzheimer disease (AD) often shows hemispherical lateral damage and reduced HbO concentrations (reflecting weakened cortical activation), especially in DLPFC and parietal cortex, through a VFT [60,64]. In spatial tasks, healthy subjects showed significant apical activation, while patients showed activation only in control tasks [64]. Studies have examined subcortical vascular dementia (SVD) patients. They found that cortical haemodynamics and metabolic response were reduced in patients with SVD in the motor cortex, and cerebral blood flow and oxygen metabolic rate were reduced, too [65]. In addition, another study used fNIRS to assess the effect of galantamine on fluency in AD patients between 4 and 8 weeks of treatment [66]. In short, these findings demonstrate the potential of fNIRS in assessing cortical oxygenation in developmental disorders and have advantages in studying children’s low-health, elderly subjects or cortical actual function-related factors.

## Conclusion

fNIRS is a highly practical and versatile imaging technology that has been widely used in many disciplines for its non-invasive, real-time, simple, bedside applications, and high practicality, especially in nerves. In the field of psychiatry, it has special significance for the monitoring and localization of blood oxygen metabolism in cerebral cortical tissue. It has been involved in many diseases and basic research. However, some shortcomings of fNIRS have been exposed in practice. For example, due to the imperfect instrument design or algorithm, the monitoring indicators in the clinical application are easy to be interfered, and the accuracy and stability are not enough. In this aspect, there is still a need for several basic types of research, such as controlling all kinds of possible interfering factors, improving hardware structure and optimizing the algorithm. In addition, the tolerance of patients in clinical practice, the control of time and the operability of the process, etc., need more accurate and comprehensive consideration in the preliminary research, so that it can be more efficient and repeatable in practical application. So far, most fNIRS studies in psychiatric disorders are about prefrontal activation, among which VFT is the most popular paradigm. At first, for the use of a few channels, to avoid the influence of the hair, the prefrontal region is a good starting point for fNIRS research, but given the recent technological development (e.g. multi-channel system), areas of interest should be expanded, especially since neuropsychiatric disorders are mostly based on alterations in distributed cerebral networks. In addition, although VFT has been shown to be reliable as a simple example, because of the differences between patients and controls, it covers only a limited aspect of neurocognitive functions, which may partly explain why the relevant cortical activation seems to change relative to the specific diagnosis. Anyhow, fNIRS is a very good analysis testing technology and is rapidly developed in the field of spectral analysis. In conclusion, with the development of detection and analysis techniques, the fNIRS will be widely applied in psychiatric and brain science research in order to provide more reliable evidence for precision diagnosis and therapeutic strategies.

## References

[1] Vigo D, Thornicroft G, Atun R. Estimating the true global burden of mental illness. The Lancet Psychiatry. 2016;3:171–178. 26851330 10.1016/S2215-0366(15)00505-2

[2] Li Y, Yu D. Variations of the functional brain network efficiency in a young clinical sample within the autism spectrum: a fNIRS investigation. Front Physiol. 2018;9:67. 29459832 10.3389/fphys.2018.00067 PMC5807729

[3] Ehlis AC, Schneider S, Dresler T, et al. Application of functional near-infrared spectroscopy in psychiatry. NeuroImage. 2014;85:478–488. 23578578 10.1016/j.neuroimage.2013.03.067

[4] Rosenbaum D, Thomas M, Hilsendegen P, et al. Stress-related dysfunction of the right inferior frontal cortex in high ruminators: an fNIRS study. J Neuroeng Rehab. 2018;18:510–517. 10.1016/j.nicl.2018.02.022 PMC5857918 29560307

[5] Akin A, Bilensoy D, Emir UE, et al. Cerebrovascular dynamics in patients with migraine: near-infrared spectroscopy study. Neurosci Lett. 2006;400:86–91. 16516381 10.1016/j.neulet.2006.02.016

[6] Faress A, Chau T. Towards a multimodal brain-computer interface: combining fNIRS and fTCD measurements to enable higher classification accuracy. NeuroImage. 2013;77:186–194. 23541802 10.1016/j.neuroimage.2013.03.028

[7] Anwar AR, Muthalib M, Perrey S, et al. Effective connectivity of cortical sensorimotor networks during finger movement tasks: a simultaneous fNIRS, fMRI, EEG study. Brain Topogr. 2016;29:645–660. 27438589 10.1007/s10548-016-0507-1

[8] Vannasing P, Cornaggia I, Vanasse C, et al. Potential brain language reorganization in a boy with refractory epilepsy; an fNIRS-EEG and fMRI comparison. Epilepsy Behav Case Rep. 2016;5:34–37. 26977406 10.1016/j.ebcr.2016.01.006 PMC4782003

[9] Huppert T, Barker J, Schmidt B, et al. Comparison of group-level, source localized activity for simultaneous functional near-infrared spectroscopy-magnetoencephalography and simultaneous fNIRS-fMRI during parametric median nerve stimulation. Neurophoton. 2017;4:015001. 10.1117/1.NPh.4.1.015001 PMC5248968 28149919

[10] Wijeakumar S, Huppert TJ, Magnotta VA, et al. Validating an image-based fNIRS approach with fMRI and a working memory task. NeuroImage. 2017;147:204–218. 27939793 10.1016/j.neuroimage.2016.12.007

[11] Bulgarelli C, Blasi A, Arridge S, et al. Dynamic causal modelling on infant fNIRS data: a validation study on a simultaneously recorded fNIRS-fMRI dataset. NeuroImage. 2018;175:413–424. 29655936 10.1016/j.neuroimage.2018.04.022 PMC5971219

[12] Yuan Z, Ye J. Fusion of fNIRS and fMRI data: identifying when and where hemodynamic signals are changing in human brains. Front Hum Neurosci. 2013;7:676. 24137124 10.3389/fnhum.2013.00676 PMC3797402

[13] Funane T, Sato H, Yahata N, et al. Concurrent fNIRS-fMRI measurement to validate a method for separating deep and shallow fNIRS signals by using multidistance optodes. Neurophoton. 2015;2:015003. 10.1117/1.NPh.2.1.015003 PMC4478864 26157983

[14] Noah JA, Ono Y, Nomoto Y, et al. fMRI validation of fNIRS measurements during a naturalistic task. J Vis Exp. 2015;100:e52116. 10.3791/52116 PMC4544944 26132365

[15] Pouliot P, Tremblay J, Robert M, et al. Nonlinear hemodynamic responses in human epilepsy: a multimodal analysis with fNIRS-EEG and fMRI-EEG. J Neurosci Methods. 2012;204:326–340. 22138633 10.1016/j.jneumeth.2011.11.016

[16] Muthalib M, Anwar AR, Perrey S, et al. Multimodal integration of fNIRS, fMRI and EEG neuroimaging. Clin Neurophysiol. 2013;124:2060–2062. 23648071 10.1016/j.clinph.2013.03.018

[17] Visani E, Canafoglia L, Gilioli I, et al. Hemodynamic and EEG time-courses during unilateral hand movement in patients with cortical myoclonus. An EEG-fMRI and EEG-TD-fNIRS study. Brain Topogr. 2015;28:915–925. 25253050 10.1007/s10548-014-0402-6

[18] Bourel-Ponchel E, Mahmoudzadeh M, Delignieres A, et al. Non-invasive, multimodal analysis of cortical activity, blood volume and neurovascular coupling in infantile spasms using EEG-fNIRS monitoring. Neuroimage Clin. 2017;15:359–366. 28580292 10.1016/j.nicl.2017.05.004 PMC5447509

[19] Al-Shargie F, Kiguchi M, Badruddin N, et al. Mental stress assessment using simultaneous measurement of EEG and fNIRS. Biomed Opt Express. 2016;7:3882–3898. 27867700 10.1364/BOE.7.003882 PMC5102531

[20] Taga G, Watanabe H, Homae F. Developmental changes in cortical sensory processing during wakefulness and sleep. NeuroImage. 2018;178:519–530. 29860079 10.1016/j.neuroimage.2018.05.075

[21] Shi J, Sakatani K, Okamoto M, et al. Correlation between LIFG and autonomic activation during stressful tasks: a functional near-infrared spectroscopy (fNIRS) study. J Huazhong Univ Sci Technol Med Sci. 2014;34:663–671. 10.1007/s11596-014-1334-9 25318875

[22] Hiraoka T, Kawashima K, Hoshino K, et al. [Neutron dosimetry intercomparison between the NIRS and institutions for fast neutron therapy in the United States of America]. Nihon Igaku Hoshasen Gakkai Zasshi. 1977;37:949–955. Japanese. 600681

[23] Zhao Q, Ji L, Jiang T. Improving performance of reflectance diffuse optical imaging using a multicentered mode. J Biomed Opt. 2006;11:064019. 17212542 10.1117/1.2400703

[24] Bhargav H, Nagendra HR, Gangadhar BN, et al. Frontal hemodynamic responses to high frequency yoga breathing in schizophrenia: a functional near-infrared spectroscopy study. Front Psychiatry. 2014;5:29. 24715879 10.3389/fpsyt.2014.00029 PMC3970016

[25] Koike S, Nishimura Y, Takizawa R, et al. Near-infrared spectroscopy in schizophrenia: a possible biomarker for predicting clinical outcome and treatment response. Front Psychiatry. 2013;4:145. 24294205 10.3389/fpsyt.2013.00145 PMC3827961

[26] Egashira K, Matsuo K, Nakashima M, et al. Blunted brain activation in patients with schizophrenia in response to emotional cognitive inhibition: a functional near-infrared spectroscopy study. Schizophr Res. 2015;162:196–204. 25595654 10.1016/j.schres.2014.12.038

[27] Aasted CM, Yucel MA, Steele SC, et al. Frontal lobe hemodynamic responses to painful stimulation: a potential brain marker of nociception. PLoS One. 2016;11:e0165226. 27806119 10.1371/journal.pone.0165226 PMC5091745

[28] Hoshi Y. Hemodynamic signals in fNIRS. Prog Brain Res. 2016;225:153–179. 27130415 10.1016/bs.pbr.2016.03.004

[29] Fallgatter AJ, Ehlis A, Wagener A, et al. Near-infrared spectroscopy in psychiatry. Nervenarzt. 2004;75:911–916. 15378251 10.1007/s00115-002-1457-2

[30] Koike S, Satomura Y, Kawasaki S, et al. Association between rostral prefrontal cortical activity and functional outcome in first-episode psychosis: a longitudinal functional near-infrared spectroscopy study. Schizophr Res. 2016;170:304–310. 26792296 10.1016/j.schres.2016.01.003

[31] Koike S, Takizawa R, Nishimura Y, et al. Different hemodynamic response patterns in the prefrontal cortical sub-regions according to the clinical stages of psychosis. Schizophr Res. 2011;132:54–61. 21813266 10.1016/j.schres.2011.07.014

[32] Okada F, Takahashi N, Tokumitsu Y. Dominance of the ‘nondominant’ hemisphere in depression. J Affect Disord. 1996;37:13–21. 8682974 10.1016/0165-0327(95)00040-2

[33] Noda T, Yoshida S, Matsuda T, et al. Frontal and right temporal activations correlate negatively with depression severity during verbal fluency task: a multi-channel near-infrared spectroscopy study. J Psychiatr Res. 2012;46:905–912. 22572569 10.1016/j.jpsychires.2012.04.001

[34] Nishizawa Y, Kanazawa T, Kawabata Y, et al. fNIRS Assessment during an emotional stroop task among patients with depression: replication and extension. Psychiatry Investig. 2019;16:80–86. 10.30773/pi.2018.11.12.2 PMC6354038 30696239

[35] Matsuo K, Kato N, Kato T. Decreased cerebral haemodynamic response to cognitive and physiological tasks in mood disorders as shown by near-infrared spectroscopy. Psychol Med. 2002;32:1029–1037. 12214784 10.1017/s0033291702005974

[36] Sato H, Obata AN, Moda I, et al. Application of near-infrared spectroscopy to measurement of hemodynamic signals accompanying stimulated saliva secretion. J Biomed Opt. 2011;16:047002. 21529092 10.1117/1.3565048

[37] Hocke LM, Duszynski CC, Debert CT, et al. Reduced functional connectivity in adults with persistent post-concussion symptoms: a functional near-infrared spectroscopy study. J Neurotrauma. 2018;35:1224–1232. 29373947 10.1089/neu.2017.5365 PMC5962910

[38] Feng K, Shen CY, Ma XY, et al. Effects of music therapy on major depressive disorder: a study of prefrontal hemodynamic functions using fNIRS. Psychiatry Res. 2019;275:86–93. 30884335 10.1016/j.psychres.2019.03.015

[39] Adorni R, Gatti A, Brugnera A, et al. Could fNIRS promote neuroscience approach in clinical psychology. Front Psychol. 2016;7:456. 27065924 10.3389/fpsyg.2016.00456 PMC4811970

[40] Zhu H, Xu J, Li J, et al. Decreased functional connectivity and disrupted neural network in the prefrontal cortex of affective disorders: a resting-state fNIRS study. J Affect Disord. 2017;221:132–144. 28645025 10.1016/j.jad.2017.06.024

[41] Schecklmann M, Dresler T, Beck S, et al. Reduced prefrontal oxygenation during object and spatial visual working memory in unipolar and bipolar depression. Psychiatry Res. 2011;194:378–384. 22079657 10.1016/j.pscychresns.2011.01.016

[42] Nishimura Y, Tanii H, Fukuda M, et al. Frontal dysfunction during a cognitive task in drug-naive patients with panic disorder as investigated by multi-channel near-infrared spectroscopy imaging. Neurosci Res. 2007;59:107–112. 17628733 10.1016/j.neures.2007.05.016

[43] Deppermann S, Vennewald N, Diemer J, et al. Does rTMS alter neurocognitive functioning in patients with panic disorder/agoraphobia? An fNIRS-based investigation of prefrontal activation during a cognitive task and its modulation via sham-controlled rTMS. Biomed Res Int. 2014;542526. 24757668 10.1155/2014/542526 PMC3976939

[44] Kawashima C, Tanaka Y, Inoue A, et al. Hyperfunction of left lateral prefrontal cortex and automatic thoughts in social anxiety disorder: a near-infrared spectroscopy study. J Affect Disord. 2016;206:256–260. 27517133 10.1016/j.jad.2016.07.028

[45] Nishimura Y, Tanii H, Hara N, et al. Relationship between the prefrontal function during a cognitive task and the severity of the symptoms in patients with panic disorder: a multi-channel NIRS study. Psychiatry Res. 2009;172:168–172. 19324535 10.1016/j.pscychresns.2009.01.001

[46] Matsuo K, Taneichi K, Matsumoto A, et al. Hypoactivation of the prefrontal cortex during verbal fluency test in PTSD: a near-infrared spectroscopy study. Psychiatry Res. 2003;124:1–10. 14511791 10.1016/s0925-4927(03)00093-3

[47] Yennu A, Tian F, Smith-Osborne A, et al. Prefrontal responses to Stroop tasks in subjects with post-traumatic stress disorder assessed by functional near infrared spectroscopy. Sci Rep. 2016;6:30157. 27452397 10.1038/srep30157 PMC4995363

[48] Ruocco AC, Medaglia JD, Tinker JR, et al. Medial prefrontal cortex hyperactivation during social exclusion in borderline personality disorder. Psychiatry Res. 2010;181:233–236. 20153143 10.1016/j.pscychresns.2009.12.001

[49] Dresler T, Schecklmann M, Ernst LH, et al. Recovery of cortical functioning in abstinent alcohol-dependent patients: prefrontal brain oxygenation during verbal fluency at different phases during withdrawal. World J Biol Psychiatry. 2012;13:135–145. 21486105 10.3109/15622975.2011.564654

[50] Schecklmann M, Schenk E, Maisch A, et al. Altered frontal and temporal brain function during olfactory stimulation in adult attention-deficit/hyperactivity disorder. Neuropsychobiology. 2011;63:66–76. 21178380 10.1159/000323448

[51] Monden Y, Dan H, Nagashima M, et al. Right prefrontal activation as a neuro-functional biomarker for monitoring acute effects of methylphenidate in ADHD children: an fNIRS study. NeuroImage Clin. 2012;1:131–140. 24179746 10.1016/j.nicl.2012.10.001 PMC3757725

[52] Gu Y, Miao S, Han J, et al. Complexity analysis of fNIRS signals in ADHD children during working memory task. Sci Rep. 2017;7:829. 28400568 10.1038/s41598-017-00965-4 PMC5429780

[53] Tamura R, Kitamura H, Endo T, et al. Decreased leftward bias of prefrontal activity in autism spectrum disorder revealed by functional near-infrared spectroscopy. Psychiatry Res. 2012;203:237–240. 22947311 10.1016/j.pscychresns.2011.12.008

[54] Yanagisawa K, Nakamura N, Tsunashima H, et al. Proposal of auxiliary diagnosis index for autism spectrum disorder using near-infrared spectroscopy. Neurophoton. 2016;3:031413. 10.1117/1.NPh.3.3.031413 PMC4900044 27335890

[55] Kenney K, Amyot F, Haber M, et al. Cerebral vascular injury in traumatic brain injury. Exp Neurol. 2016;275:353–366. 26048614 10.1016/j.expneurol.2015.05.019

[56] Plenger P, Krishnan K, Cloud M, et al. fNIRS-based investigation of the Stroop task after TBI. Brain Imaging Behav. 2016;10:357–366. 26058665 10.1007/s11682-015-9401-9

[57] Ben-David BM, van Lieshout PH, Shakuf V. Sensory source for stroop effects in persons after TBI: support from fNIRS-based investigation. Brain Imaging Behav. 2016;10:1135–1136. 26572142 10.1007/s11682-015-9477-2

[58] Kakinoki R, Duncan SFM, Ikeguchi R, et al. Motor and sensory cortical changes after contralateral cervical seventh nerve root (CC7) transfer in patients with brachial plexus injuries. J Hand Surg Asian Pac Vol. 2017;22:138–149. 28506157 10.1142/S0218810417500162

[59] Owens JA, Spitz G, Ponsford JL, et al. An investigation of white matter integrity and attention deficits following traumatic brain injury. Brain Inj. 2018;32:776–783. 29565696 10.1080/02699052.2018.1451656

[60] Wu Z, Mazzola CA, Catania L, et al. Altered cortical activation and connectivity patterns for visual attention processing in young adults post-traumatic brain injury: a functional near infrared spectroscopy study. CNS Neurosci Ther. 2018;24:539–548. 29359534 10.1111/cns.12811 PMC6490005

[61] Hibino S, Mase M, Shirataki T, et al. Oxyhemoglobin changes during cognitive rehabilitation after traumatic brain injury using near infrared spectroscopy. Neurol Med Chir. 2013;53:299–303. 10.2176/nmc.53.299 23708220

[62] Araki A, Ikegami M, Okayama A, et al. Improved prefrontal activity in AD/HD children treated with atomoxetine: a NIRS study. Brain Dev. 2015;37:76–87. 24767548 10.1016/j.braindev.2014.03.011

[63] Heinzel S, Metzger FG, Ehlis AC, et al. Aging-related cortical reorganization of verbal fluency processing: a functional near-infrared spectroscopy study. Neurobiol Aging. 2013;34:439–450. 22770542 10.1016/j.neurobiolaging.2012.05.021

[64] Metzger FG, Ehlis AC, Haeussinger FB, et al. Effects of cholinesterase inhibitor on brain activation in Alzheimer’s patients measured with functional near-infrared spectroscopy. Psychopharmacology. 2015;232:4383–4391. 26359227 10.1007/s00213-015-4066-z

[65] Hagen K, Ehlis AC, Haeussinger FB, et al. Activation during the Trail Making Test measured with functional near-infrared spectroscopy in healthy elderly subjects. NeuroImage. 2014;85:583–591. 24045079 10.1016/j.neuroimage.2013.09.014

[66] Richter MM, Herrmann MJ, Ehlis AC, et al. Brain activation in elderly people with and without dementia: influences of gender and medication. World J Biol Psychiatry. 2007;8:23–29. 17366346 10.1080/15622970600960132

